# Mildly Explosive Autoregression with Strong Mixing Errors

**DOI:** 10.3390/e24121730

**Published:** 2022-11-26

**Authors:** Xian Liu, Xiaoqin Li, Min Gao, Wenzhi Yang

**Affiliations:** School of Big Data and Statistics, Anhui University, Hefei 230039, China

**Keywords:** mildly explosive autoregression, least squares estimator, Cauchy distribution, strong mixing sequences, 62M10, 62E20

## Abstract

In this paper, we consider the mildly explosive autoregression $y_{t}=\rho_{n}y_{t-1}+u_{t}$, 1≤t≤n, where $\rho_{n}=1+c/n^{\nu }$, c>0, ν∈(0,1), and $u_{1},\ldots ,u_{n}$ are arithmetically α-mixing errors. Under some weak conditions, such as $Eu_{1}=0$, $E|u_{1}{|}^{4+\delta }<\infty$ for some δ>0 and mixing coefficients $\alpha \left(n\right)=O\left(n^{-(2+8/\delta )}\right)$, the Cauchy limiting distribution is established for the least squares (LS) estimator ${\hat{\rho }}_{n}$ of $\rho_{n}$, which extends the cases of independent errors and geometrically α-mixing errors. Some simulations for $\rho_{n}$, such as the empirical probability of the confidence interval and the empirical density, are presented to illustrate the Cauchy limiting distribution, which have good finite sample performances. In addition, we use the Cauchy limiting distribution of the LS estimator ${\hat{\rho }}_{n}$ to illustrate real data from the NASDAQ composite index from April 2011 to April 2021.

## 1. Introduction

We consider the first-order autoregressive process defined by

(1) $$y_{t}=\rho y_{t-1}+u_{t},\;1\leq t\leq n,$$

where $u_{1},u_{2},\ldots ,u_{n}$ are random errors with mean 0 and variance $\sigma^{2}>0$. It is well known that the regression coefficient ρ characterizes the properties of the process $\left\{y_{t}\right\}$. When |ρ|<1, $\left\{y_{t}\right\}$ is called a stationary process (see Brockwell and Davis [1]). For example, assume that $u_{1},\ldots ,u_{n}$ are independent and identically distributed errors with $Eu_{1}=0$, $Var\left(u_{1}\right)=\sigma^{2}$, and $E|u_{1}{|}^{2+\delta }<\infty$, with some δ>0. Then, the least squares (LS) estimator ${\hat{\rho }}_{n}$ of ρ defined by

(2) $${\hat{\rho }}_{n}=\left(\sum_{t=1}^{n}y_{t-1}y_{t}\right){\left(\sum_{t=1}^{n}y_{t-1}^{2}\right)}^{-1}$$

has a normal limiting distribution:

(3) $$\sqrt{n}\left({\hat{\rho }}_{n}-\rho \right)\overset{d}{⟶}N\left(0,1-\rho^{2}\right),$$

where |ρ|<1 (see Phillips and Magdalinos [2]). When |ρ|=1, $\left\{y_{t}\right\}$ is called a random walk process (see Dickey and Fuller [3], Wang et al. [4]). When |ρ|>1, $\left\{y_{t}\right\}$ is called an explosive process. Let $u_{1},\ldots ,u_{n}$ be independent and identically distributed Gaussian errors $N(0,\sigma^{2})$ with $\sigma^{2}>0$, and the initial condition $y_{0}=0$. White [5] and Anderson [6] showed that the LS estimator ${\hat{\rho }}_{n}$ of ρ has a Cauchy limiting distribution:

(4) $$\frac{\rho^{n}}{\rho^{2}-1}\left({\hat{\rho }}_{n}-\rho \right)\overset{d}{⟶}C,$$

where C is a standard Cauchy random variable. Moreover, let *c* be a constant, and $\rho =\rho_{n}=1+c/n^{\nu }$, where ν∈(0,1). If c<0, then $\left\{y_{t}\right\}$ is called a near-stationary process (see Chan and Wei [7]). Let $u_{1},\ldots ,u_{n}$ be independent and identically distributed errors with $Eu_{1}=0$, $Var\left(u_{1}\right)=\sigma^{2}$, and $E|u_{1}{|}^{2+\delta }<\infty$, with some δ>0. Phillips and Magdalinos [2] showed that the LS estimator ${\hat{\rho }}_{n}$ of $\rho_{n}$ has a normal limiting distribution:

(5) $$\sqrt{n^{1+\nu }}\left({\hat{\rho }}_{n}-\rho_{n}\right)\overset{d}{⟶}N\left(0,1-2c\right),$$

where c<0. If c>0, then $\left\{y_{t}\right\}$ is called a near-explosive process or mildly explosive process. Phillips and Magdalinos [2] also showed that the LS estimator ${\hat{\rho }}_{n}$ of $\rho_{n}$ has a Cauchy limiting distribution:

(6) $$\frac{n^{\nu }\rho_{n}^{n}}{2c}\left({\hat{\rho }}_{n}-\rho_{n}\right)\overset{d}{⟶}C,$$

where c>0.

It is interesting to study the near-stationary process and mildly explosive process based on dependent errors. For example, Buchmann and Chan [8] considered the near-stationary process whose errors were strongly dependent random variables; Phillips and Magdalinos [9] and Magdalinos [10] studied the mildly explosive process whose errors were moving average process with martingale differences; Aue and Horvàth [11] considered the mildly explosive process based on stable errors; Oh et al. [12] studied the mildly explosive process-based strong mixing (α-mixing) errors and obtained the Cauchy limiting distribution in (6) for the LS estimator ${\hat{\rho }}_{n}$. It is known that the sequence of α-mixing is a weakly dependent sequence. However, they assumed that $\left\{u_{n},n\geq 1\right\}$ was geometrically α-mixing, i.e., $\alpha \left(n\right)=O(|\xi {|}^{n})$ for some |ξ|<1. Obviously, it was a very strong condition. It will be more general if $\left\{u_{n},n\geq 1\right\}$ is arithmetically α-mixing, i.e., $\alpha \left(n\right)=O\left(n^{-\beta }\right)$ for some β>0. Thus, the aim of this paper is to weaken this mixing condition. We continue to investigate the mildly explosive process based on arithmetically α-mixing errors. Compared with Oh et al. [12], we use different inequalities of α-mixing sequences to prove the key Lemmas 1 and 2 (see Section 2 and Section 6). As important applications, some simulations and real data of the NASDAQ composite index from April 2011 to April 2021 are also discussed in this paper. Next, we recall the definition of α-mixing as follows:

Let N={1,2,…} and denote $F_{k}^{n}=\sigma \left(u_{t},k\leq i\leq n,i\in N\right)$ to be the σ-field generated by random variables $u_{k},u_{k+1},\ldots ,u_{n}$, 1≤k≤n. For n≥1, we define

$$\alpha \left(n\right)=\underset{m\in N}{\sup}\underset{A\in F_{1}^{m},B\in F_{m+n}^{\infty }}{\sup}\left|P\left(A\cap B\right)-P\left(A\right)P\left(B\right)\right|.$$

**Definition** **1.** *If α(n)→0 as n→∞, then $\left\{u_{n},n\geq 1\right\}$ is called a strong mixing or α-mixing sequence. If $\alpha \left(n\right)=O\left(n^{-\beta }\right)$ for some β>0, then $\left\{u_{n},n\geq 1\right\}$ is called an arithmetically α-mixing sequence. If $\alpha \left(n\right)=O(|\xi {|}^{n})$ for some |ξ|<1, then $\left\{u_{n},n\geq 1\right\}$ is called a geometrically α-mixing sequence.*

The α-mixing sequence is a weakly dependent sequence and several linear and nonlinear time series models satisfy the mixing properties. For more works on α-mixing and applications of regression, we refer the reader to Hall and Heyde [13], Györfi et al. [14], Lin and Lu [15], Fan and Yao [16], Jinan et al. [17], Escudero et al. [18], Li et al. [19], and the references therein. Many researchers have studied mildly explosive models. For example, Arvanitis and Magdalinos [20] studied the mildly explosive process under the stationary conditional heteroskedasticity errors; Liu et al. [21] investigated the mildly explosive process under the anti-persistent errors; Wang and Yu [22] studied the explosive process without Gaussian errors; Kim et al. [23] studied the explosive process without identically distributed errors. Furthermore, many researchers have used the mildly explosive model to study the behavior of economic growth and rational bubble problems, see Magdalinos and Phillips [24], Phillips et al. [25], Oh et al. [12], Liu et al. [21], and the references therein.

The rest of this paper is organized as follows. First, some conditions in Assumption (1) and two important Lemmas 1 and 2 are presented in Section 2. Consequently, the Cauchy limiting distribution for LS estimator ${\hat{\rho }}_{n}$ and the confidence interval of $\rho_{n}$ are obtained in Section 2 (see Theorem 1). We also give some remarks about the existing studies of the Cauchy limiting distribution in Section 2. As applications, some simulations on the empirical probability of the confidence interval for $\rho_{n}$ and the empirical density for $\frac{\rho_{n}^{n}}{\rho_{n}^{2}-1}\left({\hat{\rho }}_{n}-\rho_{n}\right)$ and $\frac{{\hat{\rho }}_{n}^{n}}{{\hat{\rho }}_{n}^{2}-1}\left({\hat{\rho }}_{n}-\rho_{n}\right)$ are presented in Section 3, which agree with the Cauchy limiting distribution in (6). In Section 4, the mildly explosive process is used to analyze the real data from the NASDAQ composite index from April 2011 to April 2021. It is a takeoff period of technology stocks and a faster increase in U.S. Treasury yields. Some conclusions and future research are discussed in Section 5. Finally, the proofs of main results are presented in Section 6. Throughout the paper, as n→∞, let $\overset{P}{\to }$ and $\overset{d}{\to }$ respectively denote the convergence in probability and in distribution. Let $C,C_{1},C_{2},C_{3},\cdots$ denote some positive constants not depending on *n*, which may be different in various places. If *X* and *Y* have the same distribution, we denote it as $X\overset{d}{=}Y$.

## 2. Results

We consider the mildly explosive process

(7) $$y_{t}=\rho_{n}y_{t-1}+u_{t},\;t=1,\ldots ,n,$$

where $\rho_{n}=1+c/n^{\nu }$ for some c>0, $\nu \in (\frac{\delta }{1+\delta },1)$, and δ>0. In addition, $u_{1},\ldots ,u_{n}$ are mean zeros of α-mixing errors. Some conditions in Assumption 1 are listed as follows:

**Assumption** **1.** *(A1) Let $Eu_{1}=0$ and $E|u_{1}{|}^{4+\delta }<\infty$ for some δ>0;*

*(A2) Let $\left\{u_{n},n\geq 1\right\}$ be a strictly stationarity sequence of arithmetically α-mixing with $\alpha \left(n\right)=O\left(n^{-(2+8/\delta )}\right)$, where δ is defined by (A1);*

*(A3) Let $\rho_{n}=1+c/n^{\nu }$ for some c>0, and $\nu \in (\frac{\delta }{1+\delta },1)$, where δ is defined by (A1); in addition, let $y_{0}=o_{P}\left(\sqrt{n^{\nu }}\right)$.*

In order to prove the limiting distribution of the LS estimator ${\hat{\rho }}_{n}$ of $\rho_{n}$, the normalized sample covariance $\sum_{t=1}^{n}y_{t-1}u_{t}$ can be approximated by the product of the stochastic sequences

(8) $$X_{n}=\frac{1}{\sqrt{n^{\nu }}}\sum_{t=1}^{n}\rho_{n}^{-(n-t)-1}u_{t}\;\;\mathrm{and}\;\;Y_{n}=\frac{1}{\sqrt{n^{\nu }}}\sum_{j=1}^{n}\rho_{n}^{-j}u_{j}.$$

Then, we have the following lemmas:

**Lemma** **1.** *Let the conditions (A1)–(A3) hold. Then, as n→∞,*

(9) $$\begin{array}{ccc} \frac{\rho_{n}^{-n}}{n^{\nu }}\sum_{t=1}^{n}\sum_{j=t}^{n}\rho_{n}^{t-j-1}u_{j}u_{t} & \overset{L_{2}}{⟶} & 0, \end{array}$$

(10) $$\begin{array}{ccc} \frac{\rho_{n}^{-2n+1}}{n^{\nu }}\sum_{t=1}^{n}\sum_{j=1}^{t-1}\rho_{n}^{t-j-1}u_{j}u_{t} & \overset{L_{2}}{⟶} & 0, \end{array}$$

*where $\overset{L_{2}}{⟶}$ means convergence in the mean square.*

**Lemma** **2.** *Let the the conditions (A1)–(A3) hold. Then, as n→∞, the sequences $\left\{X_{n},n\geq 1\right\}$ and $\left\{Y_{n},n\geq 1\right\}$ defined by* (8) *satisfy*

(11) $$\left(X_{n},Y_{n}\right)\overset{d}{⟶}\left(X,Y\right),$$

*where X and Y are two independent $N(0,\frac{\sigma^{2}}{2c})$ random variables with c>0 and*

(12) $$\sigma^{2}=\sum_{k=-\infty }^{\infty }Cov\left(u_{0},u_{k}\right)>0.$$

Combining this with Lemmas 1 and 2, we have the following Cauchy limiting distribution for the LS estimator ${\hat{\rho }}_{n}$ of $\rho_{n}$ as follows:

**Theorem** **1.** *Let the conditions of Lemmas 1 and 2 be satisfied. Then, as n→∞, we have*

(13) $$\begin{array}{ccc} \left(\frac{\rho_{n}^{-n}}{n^{\nu }}\sum_{t=1}^{n}y_{t-1}u_{t},\frac{\rho_{n}^{-2n}}{n^{2\nu }}\sum_{t=1}^{n}y_{t-1}^{2}\right) & \overset{d}{⟶} & (XY,Y^{2}), \end{array}$$

(14) $$\begin{array}{ccc} \frac{n^{\nu }\rho_{n}^{n}}{2c}\left({\hat{\rho }}_{n}-\rho_{n}\right) & \overset{d}{⟶} & C, \end{array}$$

*where X and Y are two independent $N(0,\frac{\sigma^{2}}{2c})$ random variables defined by* (11), *and C is a standard Cauchy random variable.*

**Remark** **1.** Let ${\left(A2\right)}^{*}$: Let $\left\{u_{n},n\geq 1\right\}$ be a strictly stationarity sequence of geometrically α-mixing; ${\left(A3\right)}^{*}$ Let $\rho_{n}=1+c/n^{\nu }$ with some c>0, ν∈(0,1), and $y_{0}=o_{P}\left(\sqrt{n^{\nu }}\right)$. Under the assumptions (A.1), ${\left(A2\right)}^{*}$, and ${\left(A3\right)}^{*}$, Oh et al. [12] considered the mildly explosive process (7) and obtained Lemmas 1 and 2 and Theorem 1, which extended Theorem 4.3 of Phillips and Magdalinos [2] based on independent errors to geometrically α-mixing errors. In order to weaken geometrically α-mixing, we use the inequalities from Doukhan and Louhichi [26] and Yang [27] to re-prove the key Lemmas 1 and 2. Thus, the mixing coefficients need to satisfy $\alpha \left(n\right)=O\left(n^{-(2+8/\delta )}\right)$ for some δ>0. For details, please the proofs of Lemmas 1 and 2 in Section 6. If positive parameter δ coming from moment condition $E|u_{1}{|}^{4+\delta }<\infty$ is large, then the mixing coefficient $\alpha \left(n\right)=O\left(n^{-(2+8/\delta )}\right)$ is weak. Similarly, if positive parameter δ is small, then the mixing coefficient $\alpha \left(n\right)=O\left(n^{-(2+8/\delta )}\right)$ becomes strong. If $\delta =\delta_{n}\to 0$, then α(n) is a geometrically decaying. So the condition $\nu \in (\frac{\delta }{1+\delta },1)$ in assumption (A3) becomes ν∈(0,1). Thus, we extend the results of Phillips and Magdalinos [2] and Oh et al. [12] to arithmetically α-mixing case. In Section 3, we give some simulations for the LS estimator ${\hat{\rho }}_{n}$ in a mildly explosive process, which agree with Theorem 1. Meanwhile, the mildly explosive model is used to analyze the data of the NASDAQ composite index from April 2011 to April 2021 in Section 4.

**Remark** **2.** *For some c>0, $\nu \in (\frac{\delta }{1+\delta },1)$, and δ>0, we take $\rho_{n}=1+c/n^{\nu }$ in* (14) *and obtain*

(15) $$\frac{n^{\nu }\rho_{n}^{n}}{2c}\left({\hat{\rho }}_{n}-\rho_{n}\right)=\frac{\rho_{n}^{n}}{2(\rho_{n}-1)}\left({\hat{\rho }}_{n}-\rho_{n}\right)\overset{d}{⟶}C$$

*and*

(16) $$\frac{\rho_{n}^{n}}{\rho_{n}^{2}-1}\left({\hat{\rho }}_{n}-\rho_{n}\right)\overset{d}{⟶}C,$$

*where it uses the fact that $\rho_{n}^{2}-1=\left(c/n^{\nu }+2\right)c/n^{\nu }∼2c/n^{\nu }=2\left(\rho_{n}-1\right)$. Here, $a_{n}∼b_{n}$ means $a_{n}/b_{n}\to 1$, as n→∞. Moreover, by Proposition A.1 of Phillips and Magdalinos [2], it has $\frac{\rho_{n}^{-n}n}{n^{\nu }}=o\left(1\right)$. Combining $n^{\nu }\rho_{n}^{n}=o\left(n\right)$ with $\frac{n^{\nu }\rho_{n}^{n}}{2c}\left({\hat{\rho }}_{n}-\rho_{n}\right)=O_{P}\left(1\right)$, we have ${\hat{\rho }}_{n}/\rho_{n}\overset{P}{⟶}1$ and ${\left({\hat{\rho }}_{n}/\rho_{n}\right)}^{n}\overset{P}{⟶}1$ (or see Oh et al. [12]). Let 0<α<1 be the significance level. Then, as in Phillips et al. [25],* (14) *in Theorem 1 suggests that a 100(1−α)% confidence interval for $\rho_{n}$ can be constructed as*

(17) $$\left[{\hat{\rho }}_{n}-\frac{{\hat{\rho }}_{n}^{2}-1}{{\hat{\rho }}_{n}^{n}}C_{\alpha },{\hat{\rho }}_{n}+\frac{{\hat{\rho }}_{n}^{2}-1}{{\hat{\rho }}_{n}^{n}}C_{\alpha }\right]:=\left[{\hat{\rho }}_{nL},{\hat{\rho }}_{nU}\right],$$

*where ${\hat{\rho }}_{nL}$ and ${\hat{\rho }}_{nU}$ are the lower bound and upper bound for $\rho_{n}$ respectively, and $C_{\alpha }$ is the two-tailed α percentile critical value of the standard Cauchy distribution. For example, $C_{0.1}=6.315$, $C_{0.05}=12.7$, and $C_{0.01}=63.65674$.*

## 3. Simulations

In this section, we conduct some simulations to evaluate the LS estimator ${\hat{\rho }}_{n}$ defined by (2). The experimental data $\left\{y_{t},t\geq 1\right\}$ are a realization from the following first-order autoregressive model

(18) $$y_{t}=\rho_{n}y_{t-1}+u_{t},\;t=1,\ldots ,n,$$

where $y_{0}=0$, $\rho_{n}=1+c/n^{\nu }$ for some c>0, $\nu \in (\frac{\delta }{1+\delta },1)$, and δ>0. In addition, $u_{1},u_{2},\ldots$ are mean zero random errors. Let the error vector ${\left(u_{1},\ldots ,u_{n}\right)}^{⊤}$ satisfy the Gaussian model such that

(19) $${\left(u_{1},\ldots ,u_{n}\right)}^{⊤}\overset{d}{=}N_{n}\left(0,\Sigma_{n}\right).$$

Here, $0=0_{n\times 1}$, and $\Sigma_{n}$ is the covariance matrix satisfying

(20) $$\Sigma_{n}={\left(\xi^{\left|i-j\right|}\right)}_{1\leq i,j\leq n}$$

for some |ξ|<1. $\Sigma_{n}$ is a positive symmetric matrix. Moreover, it is easy to check that the sequence $\{u_{n},n\geq 1$ is geometrically α-mixing. For any moment of Gaussian random variable, it is finite. Combining this with Remark 1, $\nu \in (\frac{\delta }{1+\delta },1)$ is ν∈(0,1).

Firstly, we show the simulation of the empirical probability of the confidence interval (CI) for $\rho_{n}$ defined by (17). We consider the following parameter settings

n∈{100,500,1000},c∈{0.5,1},ν∈{0.5,0.6,0.7,0.8},ξ∈{−0.3,0.3}.

The number of replications is always set at 10000 and the level of significance is 0.05. Let I(·) be the indicator function. Applying (17), we calculate the empirical probability of the true value $\rho_{n}$, i.e.,

$$\frac{1}{10000}\sum_{l=1}^{10000}I\left({\hat{\rho }}_{nL}^{\left(l\right)}\leq \rho_{n}\leq {\hat{\rho }}_{nU}^{\left(l\right)}\right),$$

where ${\hat{\rho }}_{nL}^{\left(l\right)}$ and ${\hat{\rho }}_{nU}^{\left(l\right)}$ are the two CI bounds of $\rho_{n}$ in the $l^{\mathrm{th}\;}$ replication. The results are shown in Table 1 and Table 2.

From Table 1 and Table 2, we see that the CIs under ξ=0.3 were relatively better than ξ=−0.3. It may be that the volatility of $\Sigma_{n}$ with ξ=−0.3 is relatively larger than the one with ξ=0.3. The CIs had good finite sample performance when *c* was relatively large, and ν was between 0.5 and 0.7. When c=1, it can be seen that the empirical probability was close to the nominal probability 95%.

Next, by (15), (16), ${\hat{\rho }}_{n}/\rho_{n}\overset{P}{⟶}1$, and ${\left({\hat{\rho }}_{n}/\rho_{n}\right)}^{n}\overset{P}{⟶}1$, we give some histograms to illustrate

$$\frac{\rho_{n}^{n}}{\rho_{n}^{2}-1}\left({\hat{\rho }}_{n}-\rho_{n}\right)\overset{d}{⟶}C\;\;\mathrm{and}\;\;\frac{{\hat{\rho }}_{n}^{n}}{{\hat{\rho }}_{n}^{2}-1}\left({\hat{\rho }}_{n}-\rho_{n}\right)\overset{d}{⟶}C,$$

where C is a standard Cauchy random variable. We consider the following parameter settings

n∈{500,1000,1500},c∈{0.5},ν∈{0.5},ξ∈{−0.3,0.3}.

**Figure 1:**
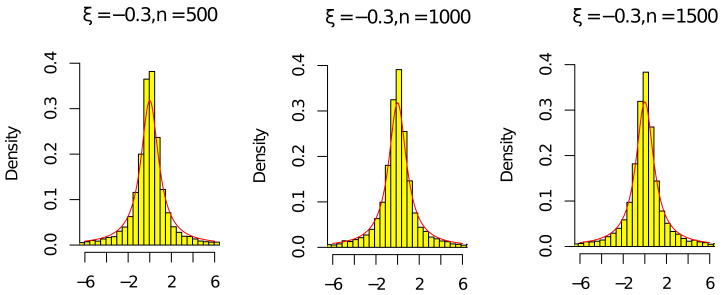
Histograms of $\frac{\rho_{n}^{n}}{\rho_{n}^{2}-1}\left({\hat{\rho }}_{n}-\rho_{n}\right)$ with ξ=−0.3 and n=[500,1000,1500].

**Figure 2:**
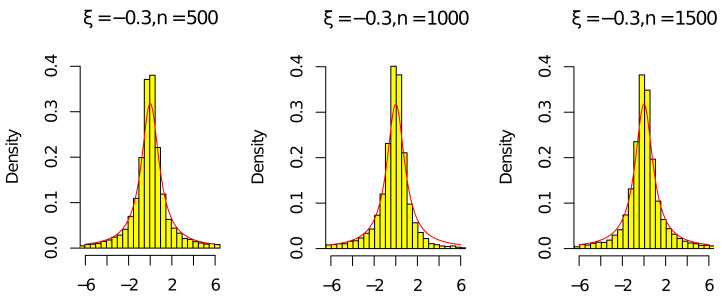
Histograms of $\frac{{\hat{\rho }}_{n}^{n}}{{\hat{\rho }}_{n}^{2}-1}\left({\hat{\rho }}_{n}-\rho_{n}\right)$ with ξ=−0.3 and n=[500,1000,1500].

**Figure 3:**
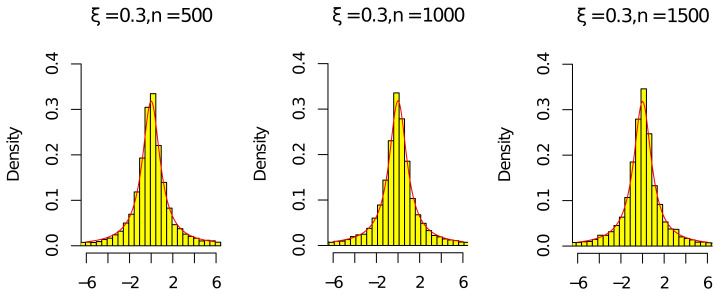
Histograms of $\frac{\rho_{n}^{n}}{\rho_{n}^{2}-1}\left({\hat{\rho }}_{n}-\rho_{n}\right)$ with ξ=0.3 and n=[500,1000,1500].

**Figure 4:**
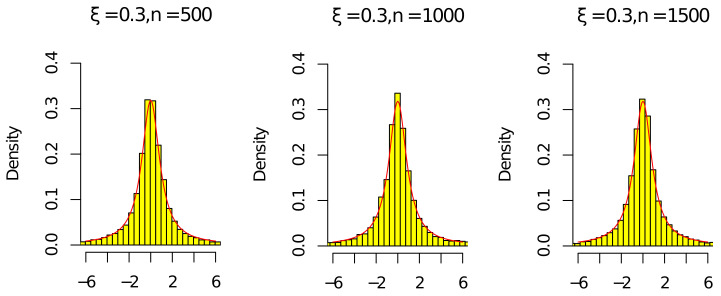
Histograms of $\frac{{\hat{\rho }}_{n}^{n}}{{\hat{\rho }}_{n}^{2}-1}\left({\hat{\rho }}_{n}-\rho_{n}\right)$ with ξ=0.3 and n=[500,1000,1500].

The red line in Figure 1, Figure 2, Figure 3 and Figure 4 is the density of the standard Cauchy random variable.

According to Figure 1, Figure 2, Figure 3 and Figure 4, the histograms $\frac{\rho_{n}^{n}}{\rho_{n}^{2}-1}\left({\hat{\rho }}_{n}-\rho_{n}\right)$ and $\frac{{\hat{\rho }}_{n}^{n}}{{\hat{\rho }}_{n}^{2}-1}\left({\hat{\rho }}_{n}-\rho_{n}\right)$ under ξ=0.3 were relatively better than the ones under ξ=−0.3 (the volatility of $\Sigma_{n}$ with ξ=−0.3 was relatively larger than the one with ξ=0.3). As the sample *n* increased, the histograms $\frac{\rho_{n}^{n}}{\rho_{n}^{2}-1}\left({\hat{\rho }}_{n}-\rho_{n}\right)$ and $\frac{{\hat{\rho }}_{n}^{n}}{{\hat{\rho }}_{n}^{2}-1}\left({\hat{\rho }}_{n}-\rho_{n}\right)$ were close to the red line of the density of the standard Cauchy random variable. Thus, the results in Figure 1, Figure 2, Figure 3 and Figure 4 and Table 1 and Table 2 agree with (14) in Theorem 1. Since the histograms with different *c* and ν were similar, we omit them here.

## 4. Real Data Analysis

**Figure 5:**
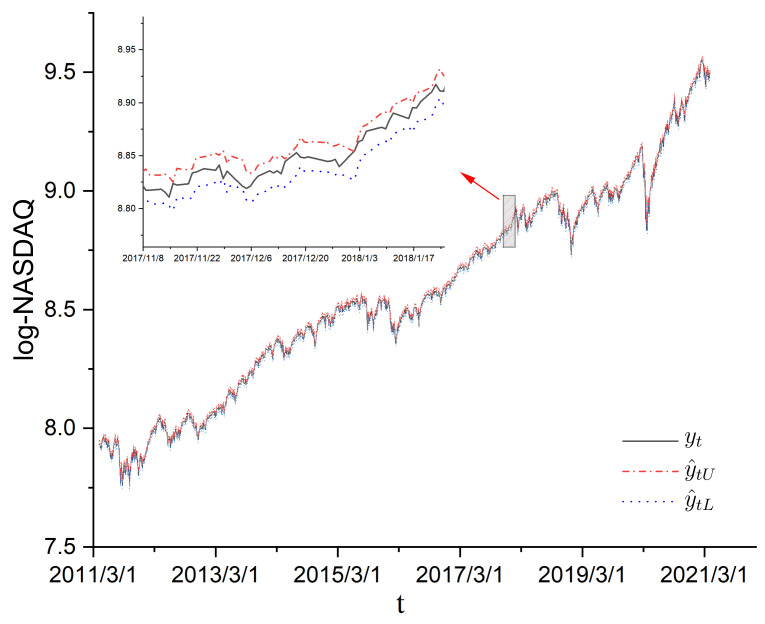
Time series for the log-NASDAQ composite index from April 2011 to April 2021.

**Figure 6:**
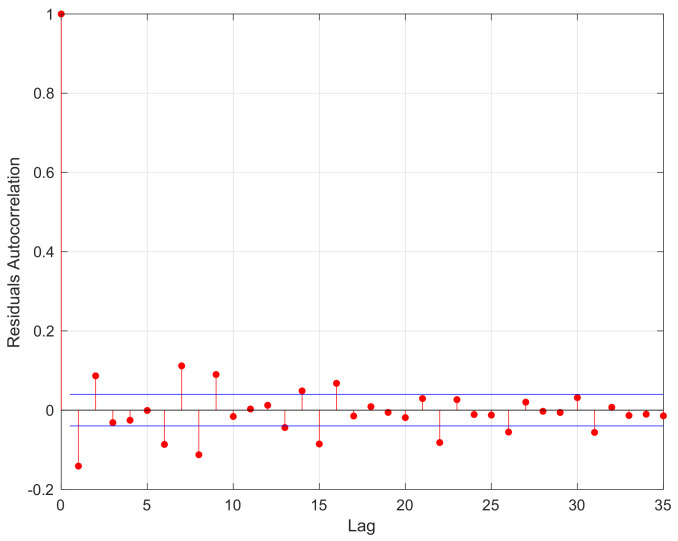
The autocorrelation coefficients based on the residuals.

In this section, we use the mildly explosive model (7) and confidence interval estimation in (17) to study the NASDAQ composite index during an inflation period. Similar to Phillips et al. [25] and Liu et al. [21], we consider the log-NASDAQ composite index for the period from April 2011 to April 2021, which contained 2522 observations denoted by $y_{t}=\log\left(P_{t}\right)$, 1≤t≤n=2522. In addition, we let $P_{0}=1$ and $y_{0}=\log\left(P_{0}\right)=0$. The scatter plots of $\left\{y_{t}\right\}$ are shown in Figure 5. According to Figure 5, the process of $\left\{y_{t}\right\}$ was increasing. Then, we used the Augmented Dickey Fuller Test (ADF Test, see [28]) to conduct the unit root test. The ADF test was −3.069 with Lag order 1, while the *p*-value of the ADF test was 0.1257. This means that the process of $\left\{y_{t}\right\}$ was nonstationary. Thus, the mildly explosive model $y_{t}=\rho_{n}y_{t-1}+u_{t}$ was considered to fit the process of $\left\{y_{t}\right\}$. We let ${\hat{u}}_{t}=y_{t}-{\hat{\rho }}_{n}y_{t-1}$ be the residuals of errors $u_{t}$, 1≤t≤n, where ${\hat{\rho }}_{n}$ is the LS estimator of $\rho_{n}$ defined by (2). Then, the residuals’ autocorrelation function (ACF) of ${\hat{u}}_{1},\ldots ,{\hat{u}}_{n}$ is shown in Figure 6.

**Figure 7:**
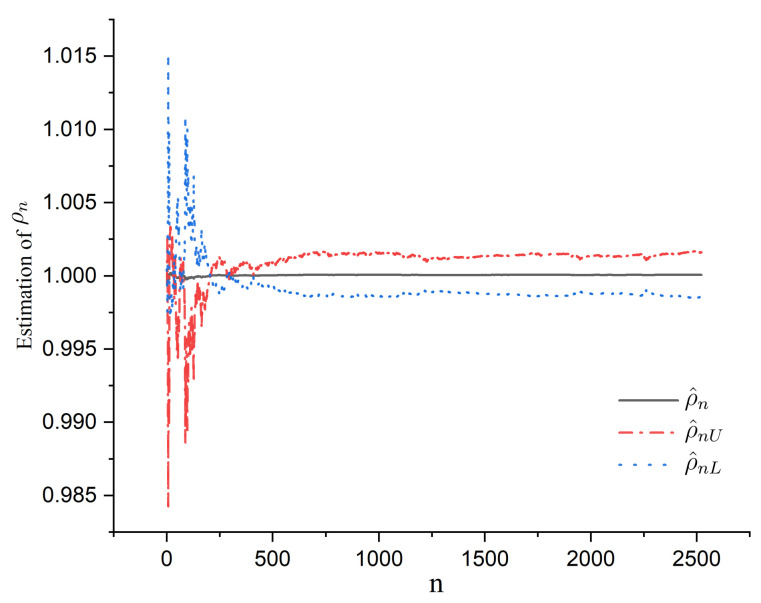
The estimators ${\hat{\rho }}_{n}$, ${\hat{\rho }}_{nL}$, and ${\hat{\rho }}_{nU}$ for $\rho_{n}$.

According to Figure 6, the autocorrelation coefficients for the residuals were around 0 as the Lag increased, which satisfied the property of α-mixing data. Then, the curves of the LS estimator ${\hat{\rho }}_{n}$ defined by (2), lower bound ${\hat{\rho }}_{nL}={\hat{\rho }}_{n}-\frac{{\hat{\rho }}_{n}^{2}-1}{{\hat{\rho }}_{n}^{n}}C_{\alpha }$ and upper bound ${\hat{\rho }}_{nU}={\hat{\rho }}_{n}+\frac{{\hat{\rho }}_{n}^{2}-1}{{\hat{\rho }}_{n}^{n}}C_{\alpha }$, for $\rho_{n}$ are also shown in Figure 7, $C_{\alpha }$ defined by (17) is the value of the standard Cauchy distribution with significance level α. With α=0.05, the curves of ${\hat{\rho }}_{n}$, ${\hat{\rho }}_{nL}$, and ${\hat{\rho }}_{nU}$ are presented in Figure 7.

According to Figure 7, the values of ${\hat{\rho }}_{n}$ approached 1 as sample *n* increased, while the lower bound ${\hat{\rho }}_{nL}$ and upper bound ${\hat{\rho }}_{nU}$ were around 1. In addition, by (17), we let ${\hat{y}}_{tL}={\hat{\rho }}_{nL}y_{t-1}$, and ${\hat{y}}_{tU}={\hat{\rho }}_{nU}y_{t-1}$, t=2,…,n. The curves of ${\hat{y}}_{tL}$ and ${\hat{y}}_{tU}$ are also shown in Figure 5. According to Figure 5, the curve $y_{t}$ was between curves ${\hat{y}}_{tL}$ and ${\hat{y}}_{tU}$, while the curve widths of ${\hat{y}}_{tL}$ and ${\hat{y}}_{tU}$ were very small. Furthermore, the period from April 2011 to April 2021 was the takeoff period of technology stocks and a faster increase in U.S. Treasury yields. Thus, these real data are a good use of the mildly explosive model and the Cauchy limiting distribution of the LS estimator in Theorem 1.

## 5. Conclusions and Discussion

The study of the mildly explosive process has received much attention from researchers, as it can be used to test the explosive behavior of economic growth. Phillips and Magdalinos [2] considered the mildly explosive process (7) based on independent errors and obtained the Cauchy limiting distribution of the LS estimator ${\hat{\rho }}_{n}$ of $\rho_{n}$. Oh et al. [12] extended Phillips and Magdalinos [2] to geometrically α-mixing errors. Obviously, the assumption of geometrically α-mixing was very strong. Thus, we considered the mildly explosive process based on arithmetically α-mixing errors. Under the condition of the mixing coefficients $\alpha \left(n\right)=O\left(n^{-(2+8/\delta )}\right)$ for some δ>0, we re-proved the key Lemmas 1 and 2. Consequently, the Cauchy limiting distribution for $\rho_{n}$ in Theorem 1 also held true. In order to illustrate the main result of the Cauchy limiting distribution, some simulations of the empirical probability of the confidence interval and the empirical density for $\rho_{n}$ were presented in Section 4. It had a good finite sample performances. As an application, we used the mildly explosive process to analyze real data from the NASDAQ composite index from April 2011 to April 2021. It was a takeoff period of technology stocks and a faster increase in U.S. Treasury yields. Moreover, it is of interest for researchers to study the random walk process, near-stationary process, mildly explosive process, and explosive process under heteroskedasticity errors (see Arvanitis and Magdalinos [20]), anti-persistent errors (see Liu et al. [21]), and other missing dependent data in the future.

## 6. Proofs of Main Results

**Lemma** **3**. *([13], Corollary A.2). Suppose that ξ and η are random variables, which are G and H-measurable, respectively, and that ${E|\eta |}^{p}<\infty$, ${E|\xi |}^{q}<\infty$, where p,q>1 and $\frac{1}{p}+\frac{1}{q}<1$. Then,*

$$\left|E\xi \eta -E\xi E\eta \right|\leq {8(E|\xi |}^{p}{)}^{1/p}{\left(E|\eta \right|}^{q}{)}^{1/q}{\left(\alpha \left(G,H\right)\right)}^{1-\frac{1}{p}-\frac{1}{q}}.$$

**Lemma** **4**. *([27], Lemma 3.2). Let ${\left\{X_{n}\right\}}_{n\geq 1}$ be an α-mixing sequence. Suppose that p and q are two positive integers. Set $\eta_{l}=\sum_{(l-1)(p+q)+1}^{(l-1)(p+q)+p}X_{j}$ for 1≤l≤k. If s>0, r>0 with $\frac{1}{s}+\frac{1}{r}=1$, then there exists a constant C such that*

$$|E\exp\left\{it\sum_{l=1}^{k}\eta_{l}\right\}-\overset{k}{\underset{l=1}{\Pi }}E\exp\left\{it\eta_{l}\right\}|\leq C|t|\alpha^{\frac{1}{s}}\left(q\right)\sum_{l=1}^{k}\left(E\right|\eta_{l}{{|}^{r})}^{1/r}.$$

**Lemma** **5**. *([27], Lemma 3.3). Let ${\left\{X_{n}\right\}}_{n\geq 1}$ be a mean zero α-mixing sequence. Let r>2. If there exist τ>0 and $\lambda >\frac{r(r+\tau )}{2\tau }$ such that $\alpha \left(n\right)=O\left(n^{-\lambda }\right)$ and $E|X_{i}{|}^{r+\tau }<\infty$, then, for any given ε>0,*

$$E|\sum_{i=1}^{n}X_{i}{|}^{r}\leq C\left\{n^{\varepsilon }\sum_{i=1}^{n}E{\left|X_{i}\right|}^{r}+{\left(\sum_{i=1}^{n}\left(E\right|X_{i}{{|}^{r+\tau })}^{\frac{2}{r+\tau }}\right)}^{\frac{r}{2}}\right\},\;\;\;\;n\geq 1,$$

*where C is a positive constant as C:=C(r,τ,λ,ε), not depending on n.*

**Proof of Lemma** **1**. It is seen that

(21) $$\begin{array}{ccc} E|\frac{\rho_{n}^{-n}}{n^{v}}\sum_{t=1}^{n}\sum_{j=t}^{n}\rho_{n}^{t-j-1}u_{j}u_{t}{|}^{2} & \leq & \frac{\rho_{n}^{-2n}}{n^{2\nu }}\sum_{t=1}^{n}\sum_{j=t}^{n}\sum_{s=1}^{n}\sum_{k=s}^{n}\rho_{n}^{-(j-t)-(k-s)-2}\left|Eu_{t}u_{j}u_{s}u_{k}\right|\\ & \leq & C_{1}\frac{\rho_{n}^{-2n}}{n^{2\nu }}\sum_{1\leq t_{1}\leq t_{2}\leq t_{3}\leq t_{4}\leq n}\left|Eu_{t_{1}}u_{t_{2}}u_{t_{3}}u_{t_{4}}\right|. \end{array}$$

Similar to Doukhan and Louhichi [26], for any q≥2, we denote

(22) $$A_{q}\left(n\right)=\sum_{1\leq t_{1}\leq \ldots \leq t_{q}\leq n}\left|Eu_{t_{1}}u_{t_{2}}\ldots u_{t_{q}}\right|$$

and

(23) $$V_{q}\left(n\right)=\sum \left|\mathrm{Cov}\left(u_{t_{1}}\ldots u_{t_{m}},u_{t_{m+1}}\ldots u_{t_{q}}\right)\right|,$$

where the sum is considered over $\left\{t_{1},\ldots ,t_{q}\right\}$ fulfilling $1\leq t_{1}\leq \ldots \leq t_{q}\leq n$ with $r=t_{m+1}-t_{m}=\max_{1\leq i<q}\left(t_{i+1}-t_{i}\right)$. Clearly,

(24) $$\begin{array}{ccc} A_{q}\left(n\right) & \leq & \sum_{1\leq t_{1}\leq \ldots \leq t_{q}\leq n}\left|Eu_{t_{1}}\ldots u_{t_{m}}Eu_{t_{m+1}}\ldots u_{t_{q}}\right|\\ & + & \sum_{1\leq t_{1}\leq \ldots \leq t_{q}\leq n}\left|\mathrm{Cov}\left(u_{t_{1}}\ldots u_{t_{m}},u_{t_{m+1}}\ldots u_{t_{q}}\right)\right|. \end{array}$$

The first term on the right-hand side of the last inequality in (24) is bounded by

(25) $$\sum_{1\leq t_{1}\leq \ldots \leq t_{q}\leq n}\left|Eu_{t_{1}}\ldots u_{t_{m}}Eu_{t_{m+1}}\ldots u_{t_{q}}\right|\leq \sum_{m=1}^{q-1}A_{m}\left(n\right)A_{q-m}\left(n\right),$$

(see [26]). Then, it follows from (24) to (25) that

(26) $$A_{q}\left(n\right)\leq \sum_{m=1}^{q-1}A_{m}\left(n\right)A_{q-m}\left(n\right)+V_{q}\left(n\right).$$

Assume that $E|u_{1}{|}^{\Delta }<\infty$ for some Δ>q, q≥2 and $\alpha \left(n\right)=O\left(n^{-\frac{\Delta q}{2(\Delta -q)}}\right)$. Then, by Lemma 3, it has

(27) $$\begin{array}{ccc} & & \left|\mathrm{Cov}(u_{t_{1}}\ldots u_{t_{m}},u_{t_{m+1}}\ldots u_{t_{q}})\right|\\ & \leq & 8\alpha^{1-\frac{q}{\Delta }}\left(r\right)[E|u_{t_{1}}\ldots u_{t_{m}}{|}^{\frac{\Delta }{m}}{]}^{\frac{m}{\Delta }}\left[E\right|u_{t_{1}}\ldots u_{t_{m}}{{|}^{\frac{\Delta }{q-m}}]}^{\frac{q-m}{\Delta }}. \end{array}$$

Using the Hölder inequality, we have that

(28) $$\begin{array}{c} \left[E\right|u_{t_{1}}\ldots u_{t_{m}}{|}^{\frac{\Delta }{m}}{]}^{\frac{m}{\Delta }}\leq \left[E\right|u_{t_{1}}{|}^{\Delta }{]}^{\frac{1}{\Delta }}\left[E\right|u_{t_{2}}\ldots u_{t_{m}}{|}^{\frac{\Delta }{m-1}}{]}^{\frac{m-1}{\Delta }}\leq \ldots \leq \left[E\right|u_{1}{{|}^{\Delta }]}^{\frac{m}{\Delta }}. \end{array}$$

Consequently, by (27) and (28),

(29) $$|\mathrm{Cov}\left(u_{t_{1}}\ldots u_{t_{m}},u_{t_{m+1}}\ldots u_{t_{q}}\right)|\leq 8\alpha^{1-\frac{q}{\Delta }}\left(r\right)\left[E\right|u_{1}{{|}^{\Delta }]}^{\frac{q}{\Delta }},$$

which implies

(30) $$M_{r,q}\sup|\mathrm{Cov}\left(u_{t_{1}}\ldots u_{t_{m}},u_{t_{m+1}}\ldots u_{t_{q}}\right)|\leq 8\alpha^{1-\frac{q}{\Delta }}\left(r\right)\left[E\right|u_{1}{{|}^{\Delta }]}^{\frac{q}{\Delta }},$$

where the supremum is taken over all $1\leq t_{1}\leq \ldots \leq t_{q}$ and 1≤m<q with $t_{m+1}-t_{m}\geq r$. By the assumption $\alpha \left(n\right)=O\left(n^{-\frac{\Delta q}{2(\Delta -q)}}\right)$, we have

(31) $$M_{r,q}=O\left(r^{-\frac{\Delta q}{2(\Delta -q)}\left(1-\frac{q}{\Delta }\right)}\right)=O\left(r^{-\frac{\Delta q}{2(\Delta -q)}\frac{\Delta -q}{\Delta }}\right)=O\left(r^{-q/2}\right).$$

In addition, by (31), we have

(32) $$V_{q}\left(n\right)\leq \sum_{t_{1}=1}^{n}\sum_{r=1}^{n-1}{\left(r+1\right)}^{q-2}M_{r,q}\leq C_{1}\sum_{t_{1}=1}^{n}\sum_{r=1}^{n-1}r^{q-2-q/2}\leq C_{2}n^{q/2}.$$

Thus, by (22)–(24), (26), (32), we obtain

(33) $$A_{q}\left(n\right)\leq \sum_{m=1}^{q-1}A_{m}\left(n\right)A_{q-m}\left(n\right)+V_{q}\left(n\right)\leq C_{4}n^{q/2}.$$

Since $E|u_{1}{|}^{4+\delta }<\infty$ with some δ>0, $\alpha \left(n\right)=O\left(n^{-(2+8/\delta )}\right)$ and the second-order stationarity of $\left\{u_{n},n\geq 1\right\}$, then the conditions $E|u_{1}{|}^{\Delta }<\infty$, Δ>q, q≥2, and $\alpha \left(n\right)=O\left(n^{-\frac{\Delta q}{2(\Delta -q)}}\right)$ for (33) are satisfied with q=4, Δ=4+δ and δ>0. Consequently, by (21) and (33), we obtain

$$\begin{array}{ccc} E|\frac{\rho_{n}^{-n}}{n^{v}}\sum_{t=1}^{n}\sum_{j=t}^{n}\rho_{n}^{t-j-1}u_{j}u_{t}{|}^{2} & \leq & C_{1}\frac{\rho_{n}^{-2n}}{n^{2\nu }}A_{4}\left(n\right)\leq C_{2}\frac{\rho_{n}^{-2n}n^{2}}{n^{2\nu }}=o\left(1\right), \end{array}$$

by using the fact that $\frac{\rho_{n}^{-n}n}{n^{\nu }}=o\left(1\right)$, i.e., for each c>0, $\rho_{n}^{-n}=o\left(n^{\nu -1}\right)$ (see Proposition A.1 of [2]). The proof of (9) is complete. On the other hand, the proof of (10) is similar, so it is omitted. □

**Proof of Lemma** **2**. By the Cramér-Wold device, it is sufficient to show that

(34) $$aX_{n}+bY_{n}\overset{d}{⟶}N\left(0,\frac{\left(a^{2}+b^{2}\right)\sigma^{2}}{2c}\right),\;\forall a,b\in R,$$

where $X_{n}$ and $Y_{n}$ are defined by (8), and $\sigma^{2}>0$ is defined by (12). Denote

$$aX_{n}+bY_{n}=\frac{1}{\sqrt{n^{\nu }}}\sum_{i=1}^{n}\xi_{ni},$$

where

$$\xi_{ni}=\left(a\rho_{n}^{-i}+b\rho_{n}^{-(n-i)-1}\right)u_{i},\;1\leq i\leq n.$$

Similar to Oh et al. [12], let $\left\{k_{n}\right\}$, $\left\{p_{n}\right\}$, and $\left\{q_{n}\right\}$ be sequences of positive integers. Then, we split the sum $\sum_{i=1}^{n}\xi_{ni}$ into large $p_{n}$ blocks and small $q_{n}$ blocks. Define $k_{n}∼n^{1-\nu /2}$, $p_{n}∼n^{\nu /2}-n^{\nu /4}$, and $q_{n}∼n^{\nu /4}$. So, we have $q_{n}/p_{n}\to 0$ and $k_{n}\left(p_{n}+q_{n}\right)/n\to 1$ as n→∞.

Denote

(35) $$\begin{array}{c} \frac{1}{\sqrt{n^{\nu }}}\sum_{i=1}^{n}\xi_{ni}=\frac{1}{\sqrt{n^{\nu }}}\sum_{m=1}^{k_{n}}y_{nm}+\frac{1}{\sqrt{n^{\nu }}}\sum_{m=1}^{k_{n}}y_{nm}^{\prime }+\frac{1}{\sqrt{n^{\nu }}}y_{nk_{n}}^{\prime \prime }, \end{array}$$

where

$$\begin{array}{cc} & y_{nm}=\sum_{i=\left(m-1\right)\left(p_{n}+q_{n}\right)+1}^{\left(m-1\right)\left(p_{n}+q_{n}\right)+p_{n}}\xi_{ni},1\leq m\leq k,\\ & y_{nm}^{\prime }=\sum_{j=\left(m-1\right)\left(p_{n}+q_{n}\right)+p_{n}+1}^{m(p_{n}+q_{n})}\xi_{nj},1\leq m\leq k,\\ & y_{nk_{n}}^{\prime \prime }=\sum_{l=k(p_{n}+q_{n})+1}^{n}\xi_{nl}. \end{array}$$

Next, we will show that

(36) $$\frac{1}{\sqrt{n^{\nu }}}\sum_{m=1}^{k_{n}}y_{nm}\overset{d}{⟶}N\left(0,\frac{\left(a^{2}+b^{2}\right)\sigma^{2}}{2c}\right)$$

and

(37) $$\frac{1}{\sqrt{n^{\nu }}}\sum_{m=1}^{k_{n}}y_{nm}^{\prime }\overset{P}{⟶}0,\;\;\frac{1}{\sqrt{n^{\nu }}}y_{nk_{n}}^{\prime \prime }\overset{P}{⟶}0.$$

Since $E|u_{1}{|}^{4+\delta }<\infty$, and $\alpha \left(n\right)=O(n^{-(2+8/\delta ))}$, we use Lemma 5 with ε=1 and obtain

(38) $$\begin{array}{ccc} & & \sum_{m=1}^{k_{n}}\left(E\right|y_{nm}{{|}^{4})}^{\frac{1}{4}}\\ & \leq & C_{1}\sum_{m=1}^{k_{n}}{\left[p_{n}\sum_{i=\left(m-1\right)\left(p_{n}+q_{n}\right)+1}^{\left(m-1\right)\left(p_{n}+q_{n}\right)+p_{n}}E{\left|\xi_{ni}\right|}^{4}+{\left(\sum_{i=\left(m-1\right)\left(p_{n}+q_{n}\right)+1}^{\left(m-1\right)\left(p_{n}+q_{n}\right)+p_{n}}\left(E\right|\xi_{ni}{{|}^{4+\delta })}^{\frac{2}{4+\delta }}\right)}^{2}\right]}^{\frac{1}{4}}\\ & \leq & C_{2}\sum_{m=1}^{k_{n}}p_{n}^{1/2}=O\left(k_{n}p_{n}^{1/2}\right). \end{array}$$

By condition (A3), (38), and Lemma 4 with r=s=2, we obtain

(39) $$\begin{array}{ccc} & & \left|E\exp\left(it\sum_{m=1}^{k_{n}}\frac{y_{nm}}{\sqrt{n^{\nu }}}\right)-\overset{k_{n}}{\underset{m=1}{\Pi }}E\exp\left(it\frac{y_{nm}}{\sqrt{n^{\nu }}}\right)\right|\\ & \leq & C_{1}t\alpha^{1/2}\left(q_{n}\right)\frac{1}{\sqrt{n^{\nu }}}\sum_{m=1}^{k_{n}}\left(E\right|y_{nm}{{|}^{2})}^{\frac{1}{2}}\\ & \leq & C_{1}t\alpha^{1/2}\left(q_{n}\right)\frac{1}{\sqrt{n^{\nu }}}\sum_{m=1}^{k_{n}}\left(E\right|y_{nm}{{|}^{4})}^{\frac{1}{4}}\\ & \leq & C_{2}\frac{1}{n^{\nu /2}}q_{n}^{-(2+8/\delta )/2}k_{n}p_{n}^{1/2}\\ & \leq & C_{3}\frac{1}{n^{\nu /2}}n^{-(1+4/\delta )\nu /4}n^{1-\nu /2}n^{\nu /4}\\ & \leq & C_{4}n^{1-\nu (1+\frac{1}{\delta })}\to 0, \end{array}$$

since $\nu >\frac{\delta }{1+\delta }$ and $\nu (1+\frac{1}{\delta })>1$.

For $m=1,2,\ldots ,k_{n}$, it can be checked that

$$\begin{array}{ccc} E(y_{nm}^{2}) & = & E{\left(\sum_{i=\left(m-1\right)\left(p_{n}+q_{n}\right)+1}^{\left(m-1\right)\left(p_{n}+q_{n}\right)+p_{n}}\left(a\rho_{n}^{-i}+b\rho_{n}^{-(n-i)-1}\right)u_{i}\right)}^{2}\\ & = & E{\left(\sum_{i=1}^{p_{n}}\left(a\rho_{n}^{-\left(m-1\right)\left(p_{n}+q_{n}\right)-i}+b\rho_{n}^{-(n-\left(m-1\right)\left(p_{n}+q_{n}\right)-i)-1}\right)u_{\left(m-1\right)\left(p_{n}+q_{n}\right)+i}\right)}^{2}\\ & = & \sum_{j=-(p_{n}-1)}^{p_{n}-1}\sum_{i=1}^{p_{n}-|j|}\left(I_{1}+I_{2}\right)Eu_{0}u_{j}, \end{array}$$

where

$$\begin{array}{ccc} I_{1} & = & a^{2}\rho_{n}^{-2\left(m-1\right)\left(p_{n}+q_{n}\right)-2i-|j|}+b^{2}\rho_{n}^{-2(n-\left(m-1\right)\left(p_{n}+q_{n}\right)-i)+|j|-1},\\ I_{2} & = & ab\rho_{n}^{-n-1}\left(\rho_{n}^{-|j|}+\rho_{n}^{|j|}\right). \end{array}$$

Similar to Oh et al. [12], there is $\sum_{j=-(p_{n}-1)}^{p_{n}-1}\rho_{n}^{-|j|}Eu_{0}u_{j}\to \sigma^{2}>0$, where $\sigma^{2}$ is defined by (12). Combining this with $\frac{\rho_{n}^{-n}n}{n^{\nu }}=o\left(1\right)$ and $k_{n}p_{n}∼n$, we establish that

(40) $$\begin{array}{ccc} \sum_{m=1}^{k_{n}}E{\left(\frac{y_{nm}}{\sqrt{n^{\nu }}}\right)}^{2} & = & \frac{1}{n^{\nu }}\sum_{m=1}^{k_{n}}\sum_{j=-(p_{n}-1)}^{p_{n}-1}\sum_{i=1}^{p_{n}-|j|}\left(I_{1}+I_{2}\right)Eu_{0}u_{j}\\ & = & \sum_{m=1}^{k_{n}}\frac{a^{2}\rho_{n}^{-2\left(m-1\right)\left(p_{n}+q_{n}\right)+2}}{n^{\nu }\left(\rho_{n}^{2}-1\right)}\sum_{j=-(p_{n}-1)}^{p_{n}-1}\rho_{n}^{-|j|}\left(1-\rho_{n}^{-2(p_{n}-|j|)}\right)Eu_{0}u_{j}\\ & + & \sum_{m=1}^{k_{n}}\frac{b^{2}\rho_{n}^{-2(n-\left(m-1\right)\left(p_{n}+q_{n}\right))+2}}{n^{\nu }\left(\rho_{n}^{2}-1\right)}\sum_{j=-(p_{n}-1)}^{p_{n}-1}\rho_{n}^{|j|}\left(\rho_{n}^{2(p_{n}-|j|)}-1\right)Eu_{0}u_{j}\\ & \to & \frac{\left(a^{2}+b^{2}\right)\sigma^{2}}{2c}. \end{array}$$

Meanwhile, for all η>0, by (38), (40), and the Markov inequality, we have

$$\begin{array}{ccc} E[{\left(\frac{y_{nm}}{\sqrt{n^{\nu }}}\right)}^{2}I(|\frac{y_{nm}}{\sqrt{n^{\nu }}}\left|>\eta )\right] & = & \frac{1}{n^{\nu }}E[y_{nm}^{2}I(|\frac{y_{nm}}{\sqrt{n^{\nu }}}\left|>\eta )\right]\\ & \leq & \frac{1}{\eta^{2}n^{2\nu }}{\left(Ey_{nm}^{4}\right)}^{\frac{1}{2}}P(|y_{nm}{|}^{2}>n^{\nu })\\ & \leq & \frac{cp_{n}}{\eta^{2}n^{2\nu }}Ey_{nm}^{2}=O\left(p_{n}^{2}/n^{2\nu }\right), \end{array}$$

which implies

(41) $$\begin{array}{ccc} & & \sum_{m=1}^{k_{n}}E[{\left(\frac{y_{nm}}{\sqrt{n^{\nu }}}\right)}^{2}I(|\frac{y_{nm}}{\sqrt{n^{\nu }}}\left|>\eta )\right]=O\left(k_{n}p_{n}^{2}/n^{2\nu }\right)=o\left(1\right). \end{array}$$

Consequently, (36) follows from (39), (40), and (41) immediately.

On the other hand, similar to the proof of (36), we have

(42) $$\begin{array}{c} \frac{1}{\sqrt{n^{\nu /2}}}\sum_{m=1}^{k_{n}}y_{nm}^{\prime }\overset{d}{⟶}N\left(0,\frac{\left(a^{2}+b^{2}\right)\sigma^{2}}{2c}\right), \end{array}$$

which implies

(43) $$\begin{array}{c} \frac{1}{\sqrt{n^{\nu }}}\sum_{m=1}^{k_{n}}y_{nm}^{\prime }\overset{P}{⟶}0. \end{array}$$

Furthermore, by the proof of (38) and $k_{n}\left(p_{n}+q_{n}\right)/n\to 1$, we obtain

(44) $$\begin{array}{ccc} E{\left(\frac{1}{\sqrt{n^{\nu }}}y_{nk_{n}}^{\prime \prime }\right)}^{2} & = & \frac{1}{n^{\nu }}E{\left(y_{nk}^{\prime \prime }\right)}^{2}\leq \frac{1}{n^{\nu }}{\left(E{\left(y_{nk}^{\prime \prime }\right)}^{4}\right)}^{1/2}\\ & \leq & \frac{C}{n^{\nu }}{\left(\sum_{l=k_{n}\left(p_{n}+q_{n}\right)+1}^{n}a\rho_{n}^{-l}+b\rho_{n}^{-(n-l)-1}\right)}^{2}=o\left(1\right). \end{array}$$

Thus, by (43) and (44), the proof of (37) is complete. Combining (35), (36) and (37), we obtain the result of (34). □

**Proof of Theorem** **1**. Similar to the proofs of Theorem 4.3 of Phillips and Magdalinos [2] and Theorem 1 of Oh et al. [12], by Lemmas 1 and 2, it is easy to obtain the results (13) and (14). We omit the details here. □

## Figures and Tables

**Table 1:** Empirical probability of 95% CI of $\rho_{n}$ with ξ=−0.3.

ν	n=100		n=500		n=1000	
	*c* = 0.5	*c* = 1	*c* = 0.5	*c* = 1	*c* = 0.5	*c* = 1
	CI	CI	CI	CI	CI	CI
0.5	0.9501	0.9777	0.9629	0.9721	0.9611	0.9733
0.6	0.9405	0.9681	0.9508	0.9637	0.9582	0.9748
0.7	0.9433	0.9687	0.9464	0.9613	0.9248	0.9493
0.8	0.9464	0.9481	0.9323	0.9394	0.9208	0.9293

**Table 2:** Empirical probability of 95% CI of $\rho_{n}$ with ξ=0.3.

ν	n=100		n=500		n=1000	
	*c* = 0.5	*c* = 1	*c* = 0.5	*c* = 1	*c* = 0.5	*c* = 1
	CI	CI	CI	CI	CI	CI
0.5	0.9472	0.9541	0.9572	0.9624	0.9536	0.9571
0.6	0.9371	0.9592	0.9569	0.9576	0.9522	0.9587
0.7	0.9724	0.9498	0.9476	0.9554	0.9619	0.9515
0.8	0.9972	0.9721	0.9919	0.9475	0.9961	0.9536

## Data Availability

Not applicable.
